# Urine proteome analysis by C_18_ plate–matrix-assisted laser desorption/ionization time-of-flight mass spectrometry allows noninvasive differential diagnosis and prediction of diabetic nephropathy

**DOI:** 10.1371/journal.pone.0200945

**Published:** 2018-07-19

**Authors:** Chao-Jung Chen, Wen-Ling Liao, Chiz-Tzung Chang, Hsin-Yi Liao, Fuu-Jen Tsai

**Affiliations:** 1 Proteomics Core Laboratory, Department of Medical Research, China Medical University Hospital, Taichung, Taiwan; 2 Graduate Institute of Integrated Medicine, China Medical University, Taichung, Taiwan; 3 Center for Personalized Medicine, China Medical University Hospital, Taichung, Taiwan; 4 College of Medicine, China Medical University, Taichung, Taiwan; 5 Division of Nephrology, China Medical University Hospital, Taichung, Taiwan; 6 Department of Health and Nutrition Biotechnology, Asia University, Taichung, Taiwan; 7 School of Chinese Medicine, China Medical University, Taichung, Taiwan; 8 Department of Medical Genetics, China Medical University Hospital, Taichung, Taiwan; International University of Health and Welfare, School of Medicine, JAPAN

## Abstract

Diabetic nephropathy (DN) is one of the most common complications in diabetic patients. New noninvasive markers are still needed for the early detection of DN before identifiable alternations in kidney function or urine albumin excretion occurs. A C_18_ plate and matrix-assisted laser desorption/ionization time-of-flight mass spectrometry (MALDI-TOF-MS) were used to compare the urinary protein profiles of 238 subjects from the following 4 groups: patients with type 2 diabetic (T2D) with microalbuminuria, patients with DM without micro- or macroalbuminuria, patients with micro- or macroalbuminuria due to nondiabetic disease, and healthy controls. β2-microglobulin (B2M) and Clara-cell protein (CC16) were found to be highly released in the urine of patients with proteinuria due to nondiabetic or diabetic diseases. In differentiating nephropathy from healthy subject, the B2M and CC16 markers have a combined sensitivity and specificity of 77.3% and 91.8%, respectively. In distinguishing T2D with microalbuminuria from T2D patients, the combined markers have sensitivity and specificity of 66% and 73%, respectively. The predictive ability of B2M and CC16 for early renal functional decline (ERFD) was validated in 125 T2D patients with a follow-up times. The odds ratio (OR) of combined B2M and CC16 markers for developing ERFD was 7.59 (95% CI: 1.97–29.24). The detection of B2M and CC16 with the C_18_ plate—MALDI-TOF MS approach could be an attractive and practical assay for rapid diagnosis of nephropathy in nondiabetic/diabetic patients and as a predictor of ERFD among T2D patients who had not manifested significant kidney disease at baseline.

## 1. Introduction

Diabetic nephropathy (DN) is one of the most common complications in diabetic patients. Renal disease develops in approximately 20–40% of type 2 diabetic (T2D) patients [1]. In addition, DN is the leading cause of end-stage renal disease (ESRD). Microalbuminuria (urine albumin excretion 30–300 mg/24 h) is the first sign of kidney dysfunction because it can progress to macroalbuminuria (>300 mg/24 h) and subsequently to kidney failure [2, 3]. However, because microalbuminuria may be induced by comorbidity factors, such as preexisting glomerulonephritis, viral hepatitis infection, nephrotoxic agent usage, cardiovascular events, heart failure, or some systemic autoimmune diseases, it has a lower predictive value for DN [4]. Therefore, new noninvasive markers are needed for the early detection of DN before identifiable alternations in kidney function or urine albumin excretion occurs.

Because urine sampling is noninvasive and the protein composition in urine is more relevant to kidney function, urine is a suitable sample source for discovering protein biomarkers of type 2 diabetic nephropathy (T2DN). Mass spectrometry (MS)-based proteomics, an emerging approach for analyzing the global protein content of a biological sample, has been widely applied to the search of novel biomarkers of diseases [5–8]. To avoid a high-salt content in urine (which may interfere with MS signals), or reduce protein complexity, the urine sample is usually desalted or purified prior to MS detection. By binding the proteins onto a solid-phase chromatography plate, protein samples can be rapidly purified and subjected to TOF-MS analysis. Using such surface-enhanced laser desorption/ionization time-of-flight mass spectrometry (SELDI-TOF-MS, a variation of MALDI-TOF-MS) for the rapid profiling of intact protein, it was found that UbA52 and β_2_-microglobulin (B2M) were significantly excreted in the urine of diabetic patients with macro- or microalbuminuria [1]. Papale et al. [9], using SELDI-TOF MS with a CM10 protein purification chip, also found that B2M and ubiquitin were highly excreted in DN. Wu et al. [10]., using SELDI-TOF MS with a hydrophobic purification chip (H50 chip), found that a 4-peak panel of *m/z* 2797.3, 4545.77, 4984.03, and 9083.71 could be used as biomarkers for T2DN.

However, SELDI-TOF MS is limited by its high-cost protein purification chips and specified TOF MS. In order to provide a low-cost and universal protein purification chip, we have developed a C_18_ plate that allows for rapid sample desalting and protein purification processes [11].

In this study, a C_18_ plate coupled with MALDI-TOF MS detection was used for the analysis of urine protein patterns in healthy controls, non-diabetic subjects with kidney injury, and type 2 diabetic patients with and without microalbuminuria. We successfully identified 2 protein biomarkers, β2-microglobulin (B2M) and Clara-cell protein (CC16), and established a rapid and low-cost diagnostic MALDI-TOF MS platform for the early detection of T2DN. We also performed a cohort study to validate the predictive ability of B2M and CC16 on C18 plate/MALDI-TOF, in order to predict the early renal functional decline (ERFD) among T2D patients in the Taiwanese population.

## 2. Materials and methods

Polydimethylsiloxane (PDMS) prepolymer was purchased from Dow Corning (Sylgard 184Midland, MI, USA). Acetonitrile (ACN) and trifluoroacetic acid (TFA) were purchased from J.T. Baker (Phillipsburg, NJ, USA). Dithiothreitol (DTT), iodoacetamide (IAA), and formic acid (FA) were purchased from Sigma-Aldrich(St Louis, MO, USA). Octadecyl-coated silica particles (C_18_, 3 μm, 100 Å, Develosil) were purchased from Nomura Chemical Co., Ltd (Seto, Japan). Sinapic acid (SA) was purchased from Bruker Daltonics (Germany). Trypsin (modified, sequencing grade) was obtained from Promega (Madison, WI, USA). Urea, was purchased from Bio Basic Inc. (Toronto, Canada).

### 2.1 Study population and samples

A cross-sectional study design was used for the discovery and validation of protein markers by C18 plate/MALDI-TOF MS. The study protocol, including sample collection, preparation, and analysis, was approved by the local ethics committee of the China Medical University Hospital, Taichung, Taiwan, and performed according to the principles of the Declaration of Helsinki. All subjects (n = 238) had given their informed consent before the study. The following 4 groups were defined according to clinical course and urinary albumin excretion levels: patients with DM with microalbuminuria (DM-NP; n = 53, 30 < albumin-to-creatinine (ACR) ratio < 300 mg/g), patients with DM without micro- or macroalbuminuria (DM-WNP; n = 87, ACR < 30 mg/g), patients with micro- or macroalbuminuria due to nondiabetic disease (WDM-NP; n = 48, 30 mg/g < ACR), and healthy controls (n = 50; ACR < 30 mg/g). The clinical characteristics of the 4 groups are shown in Table 1.

**Table 1:** Clinical and biochemical parameters for the healthy, WDM-NP, DM-WNP, and DM-NP subjects.

	Unit	Healthy (n = 50)	WDM-NP (n = 48)	DM-WNP (n = 87)	DM-NP (n = 53)
**Gender (M/F)**	None	23/27	19/29	47/40	27/26
**Age**	years	51.6 (7.6)	64.3 (15.3)	61.1 (11.0)	65.5 (11.5)
**BMI**	kg/m^2^	23.5 (3.5)	25.2 (5.3)	25.0 (3.2)	26.2 (4.0)
**HbA1c**	%	5.5 (0.4)	5.8 (0.4)	6.6 (0.5)	6.9 (0.6)
**Creatinine**	mg dL^-1^	1.0 (0.2)	1.2 (1.4)	0.80 (0.20)	0.80 (0.20)
**eGFR**	ml/min	76.0 (10.7)	67.1 (28.0)	92.8 (22.7)	88.6 (24.0)
**Albuminuria**	mg dL^-1^	0.7 (0.5)	534.6 (980.0)	7.1(8.1)	85.9(103.1)
**Urine creatinine**	mg dL^-1^	118.7(66.2)	141.9 (228.9)	90.3 (59.3)	78.7 (53.3)
**Urine ACR**	mg/g	6.3 (4.0)	961.1 (1911.5)	8.1 (6.9)	106.1 (70.4)

Values are expressed as the mean ± standard deviation. WDM-NP group, patients with micro- or macroalbuminuria due to nondiabetic disease; DM-WNP group, patients with diabetes mellitus (DM) without micro- or macroalbuminuria; DM-NP, patients with DM with microalbuminuria; BMI, body mass index; eGFR, estimated glomerular filtration rate; ACR, albumin-to-creatinine ratio.

### 2.2 Study population for follow-up verification

A total of 125 T2D subjects, including 56 had ERFD (case) and 69 did not have ERFD (control) were included in this nested case-control study. All patients had normal renal function (estimated glomerular filtration rate [eGFR] > 60 mL/min per 1.73 m^2^ and an ACR < 300 mg/g) at the time of enrollment. Among these participants, 56 had ERFD, and 69 did not have ERFD during follow-up. ERFD is defined as having more than 3.3 mL/min per 1.73 m^2^ decline in eGFR per year [12]. The Modified Diet in Renal Disease (MDRD) equation [13] was used to estimate GFR.

### 2.3 Urine sample preparation for protein profiling

Midstream urine was collected in a 15-mL centrifuge tube for protein sampling. To reduce the protein degradation effect, 500 μL of a protease inhibitor cocktail solution (1 protease inhibitor tablet dissolved in 10 mL of double-distilled water (ddH_2_O)) was added to 10 mL of each collected urine sample. The urine samples were centrifuged for 20 min at 3000 *g* and 4 °C. After elimination of the precipitate, the supernatant was collected for use immediately or stored at –80 °C.

### 2.4 Protein desalting by C_18_ plate and protein profiling by MALDI-TOF MS

**Fig 1:**
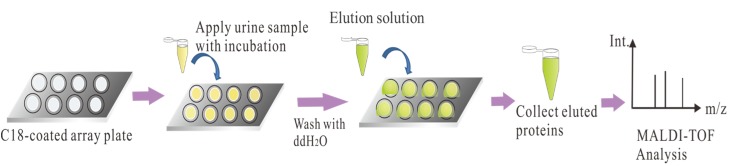
Analytical flowchart of urinary proteins by C18 plate-MALDI-TOF approach. Urine samples were from healthy, DM-WNP, DM-NP or WDM-NP subjects.

The C_18_ plates were fabricated according to our previous study [11] and the analytical flowchart of urinary protein analysis was shown in Fig 1. The C_18_ spots were first washed with a 100% MeOH solution to remove contaminants and nonspecifically adsorbed compounds. The urine sample (20 μL) was directly loaded onto the C_18_ spots and incubated for 10 min or until they had dried. The spots were then washed with ddH_2_O to remove salts. The desalted proteins were eluted from the C_18_ plate using 3 μL of an 80% ACN/0.1% TFA solution. For MALDI-TOF MS analysis, the eluted proteins were mixed with 2 μL of SA solution (saturated SA in 30% ACN/0.1% TFA) on a MALDI-target. After SA/protein co-crystallization, the MALDI-target was analyzed with a MALDI-TOF/TOF MS system (Ultraflex III TOF/TOF; Bruker Daltonics) equipped with a Smartbeam laser system, using the linear mode.

### 2.5 Purification of protein marker peaks

A liquid chromatography (LC) pumping system (Ultimate 3000; Dionex) equipped with an LC column (XBridge Protein BEH C_4_ column, 300 Å, 3.5 μm, 2.1 mm × 150 mm; Waters) was used for purifying the protein. The mobile phases were solvent A (5% ACN and 0.1% FA) and solvent B (100% ACN and 0.1% FA). Gradient elution at a flow rate of 250 μL/min was set as follows: 1% B for 1.5 min, 1% to 30% B over 1 min, 30% to 80% B over 16 min, 80% B for 2 min, and 80% B to 1% B over 5 min. The eluents were monitored with a UV detector (VWD-3400 RS; Dionex) at the wavelengths of 220 and 280 nm. The eluents were collected at 60-s intervals. Each fraction was analyzed by MALDI-TOF MS to confirm the successful purification of the marker peak at *m/z* ~15860. The purified protein subfraction with the marker peak at *m/z* 15860 and its neighboring subfractions (as control subfractions) were dried in a centrifugal concentrator (miVac Duo Concentrator; Genevac, NY, USA) and then subjected to in-solution digestion and nanoLC-MS/MS analysis for identification.

### 2.6 In-solution digestion

The purified protein marker peak at *m/z* 15800 was re-dissolved in 4 M urea and reduced with 10 mM DTT for 45 min at 37 °C. Then, 55 mM IAA was added and the mixture was incubated for 60 min in the dark at 25 °C. Ammonium bicarbonate buffer (10 mM) was added to the protein solution to reduce the urea concentration to below 1 M. Trypsin was then added to the protein solution at an enzyme-to-substrate ratio of 1:25 (w/w) for 16 h at 37 °C. The peptide solution was desalted with C_18_ Z-tips, dried in a centrifugal concentrator, and then reconstituted with 10 μL of 0.1% FA for nanoLC-MS/MS analysis.

### 2.7 NanoLC-MS/MS analysis

NanoLC-MS/MS was performed with a nanoflow ultra-performance liquid chromatography system (UltiMate 3000 RSLCnano system; Dionex) coupled to a hybrid quadrupole time-of-flight (Q-TOF) mass spectrometer (maXis Impact; Bruker). After sample loading, the peptides were eluted frim a trap column into an analytical column (Acclaim PepMap C_18_, 2 μm, 100 Å, 75 μm × 250 mm; Thermo Scientific) coupled to a nano-electrospray ionization source on the Q-TOF mass spectrometer. A gradient elution of 8% ACN (0.1% FA) to 40% ACN (0.1% FA) over 36 min was used at a flow rate of 300 nL/min for tryptic peptide separation. Eight precursors of charge +2, +3, and +4 from each TOF MS scan were dynamically selected and isolated for MS/MS fragment ion scanning. The MS and MS/MS accumulation were set at 1 and 10 Hz, respectively.

### 2.8 Protein database search

The spectra acquired by nanoLC-MS/MS were converted into xml files using DataAnalysis (version 4.1; Bruker) and searched against the Swissprot (release 51.0) database using MASCOT (version 2.2.07). The MASCOT search parameters for precursor ion and fragment ion tolerance were 80 ppm and 0.07 Da, respectively. The following search parameters were selected: Taxonomy, Human; missed cleavages, 1; enzyme, trypsin; fixed modifications, carbamidomethyl (C); and variable modifications, oxidation (M) and deamidation (NQ). Peptides were considered as “identified” if their individual MASCOT ion score was higher than 25 (*p* < 0.01).

### 2.9 ELISA measurement of B2M and Clara-cell protein in urine

The urine B2M and Clara-cell protein (CC16) concentrations were measured by ELISA using commercial kits (Cloud-Clone Corp.) according to the manufacturer’s instructions. All samples were processed using the same equipment and by the same laboratory technician, who was blinded to all clinical data. The Mann-Whitney test was used to compare differences in the medium values, which were expressed as the medium with quartile values (25%, 75%).

### 2.10 Statistical analysis

Continuous data were presented as means and standard deviations or medians and interquartile ranges, and categorical data were presented as proportions. Two independent sample T-test was used for comparisons of means of continuous variables, and chi-squared test was used for comparisons of the frequencies of categorical variables between groups. The association between potential urinary biomarkers and ERFD was estimated using logistic regression model, and odd ratios (ORs) and 95% confidence intervals (CIs) were calculated. The receiver operating characteristic (ROC) curve was constructed to determine the sensitivity and specificity of the potential biomarker. Statistical analyses were conducted using SigmaPlot 11.1 (Systat Software Inc., CA, USA) and SPSS statistical software, 22.0 (IBM Corp., NY, USA). The *p* values of less than 0.05 (two-sided) were considered significant.

## 3. Results

### 3.1 Urine sample preparation by C_18_ plate

**Fig 2:**
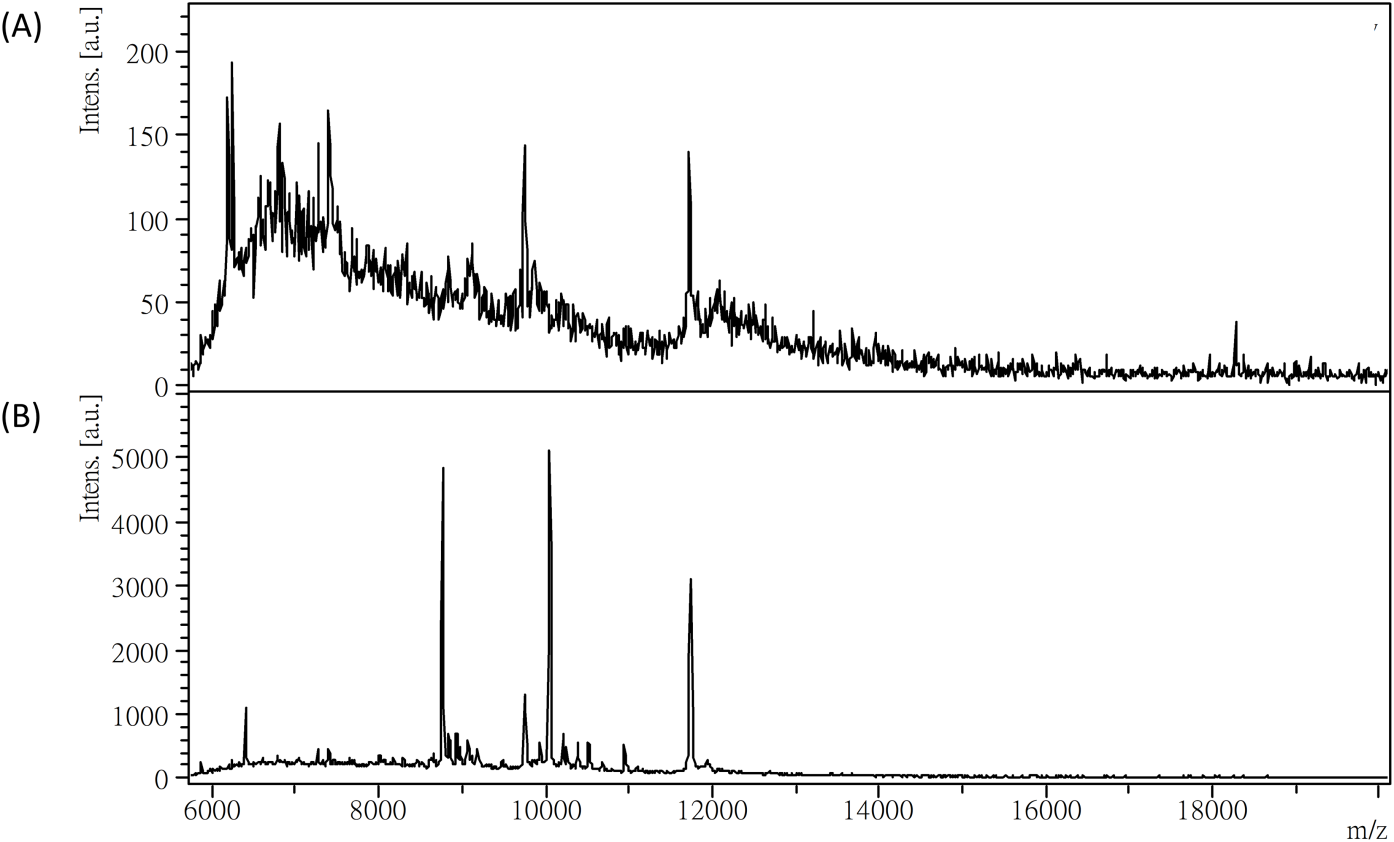
MALDI-TOF mass spectra of urine samples. (A) Without desalting. (B) With C_18_-plate desalting.

A high salt content in urine could interfere with MALDI crystallization and result in poor MS signals. To evaluate the salt effect on the urinary protein profiling, 20 μL of urine was directly applied to MALDI-TOF MS analysis without desalting. The resulting protein profile with poor signals is shown in Fig 2A. To avoid the salt interference effect, we used a hydrophobic C_18_ plate to remove salts but retain proteins in the urine samples. As shown in Fig 2B, after applying the desalted urine sample to the C_18_ plate, the protein signals were greatly improved.

To evaluate the minimum protein amount required for acquiring a protein profile in this study, different urine protein amounts were tested. Similar protein profiles were obtained when 0.6–2.38 μg amounts were applied (S1 Fig). The protein concentrations (expressed as the medium with quartile values (25%, 75%)) measured by Bradford protein assay in urine samples from the healthy, WDM-NP, DM-WNP, and DM-NP groups were 0.06 μg/μL (0.03–0.09 μg/μL), 0.34 μg/μL (0.13–1.11 μg/μL), 0.05 μg/μL (0.03–0.08 μg/μL), and 0.13 μg/μL (0.10–0.19 μg/μL), respectively. (S2 Fig) Therefore, the protein amount in 20 μL of urine sample was sufficient to give an informational protein profile in this study.

### 3.2 Protein excretion patterns in normal and pathological urines

**Fig 3:**
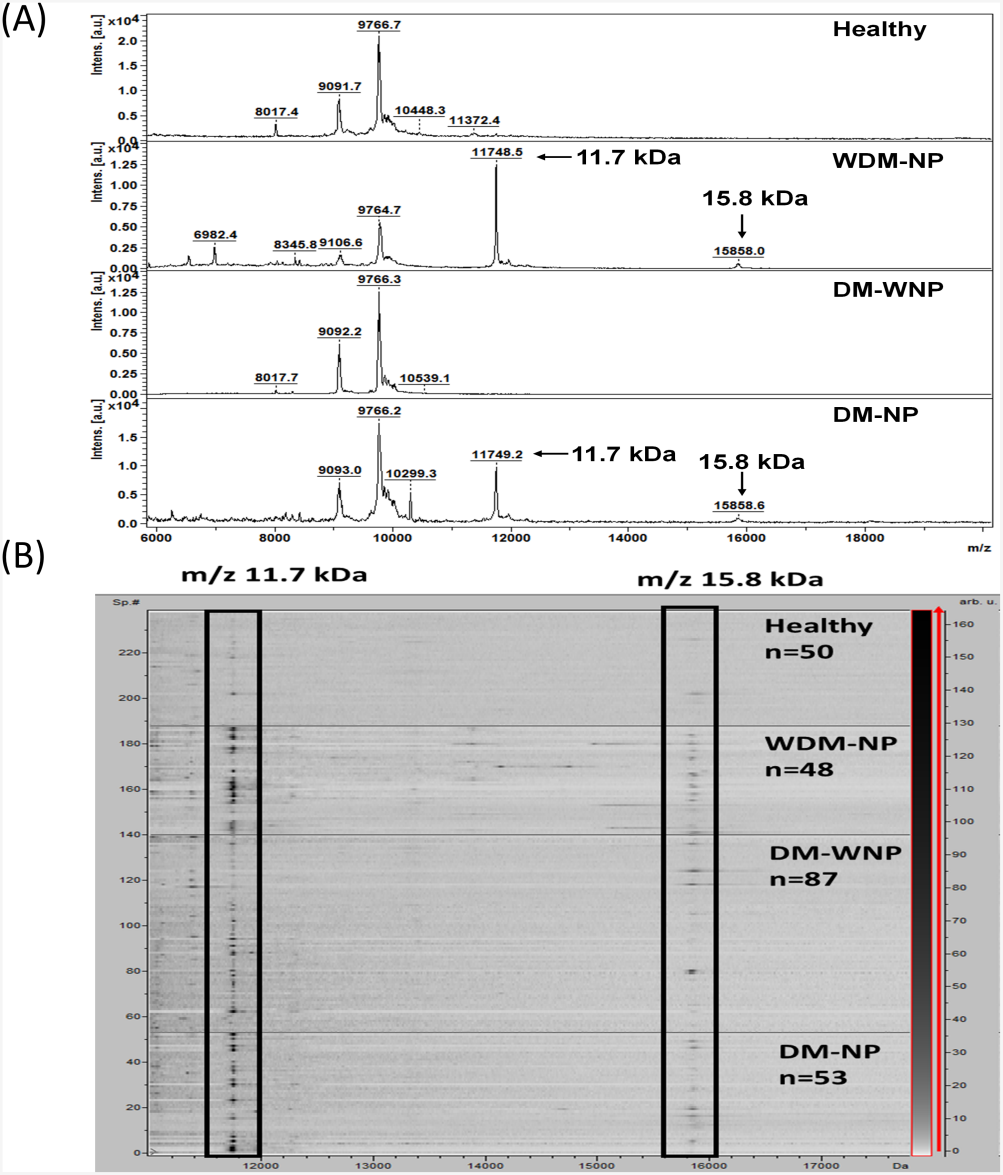
(A) Representative MALDI-TOF mass spectra of urine samples from a healthy individual and patients with WDM-NP, DM-WNP and DM-NP. (B) Pseudo gel view of the urine protein profiles. The horizontal line indicates the separation between the healthy, WDM-NP, DM-WNP, and DM-NP groups. The differential density along the x-axis represents the abundance of specific protein in the samples.

**Fig 4:**
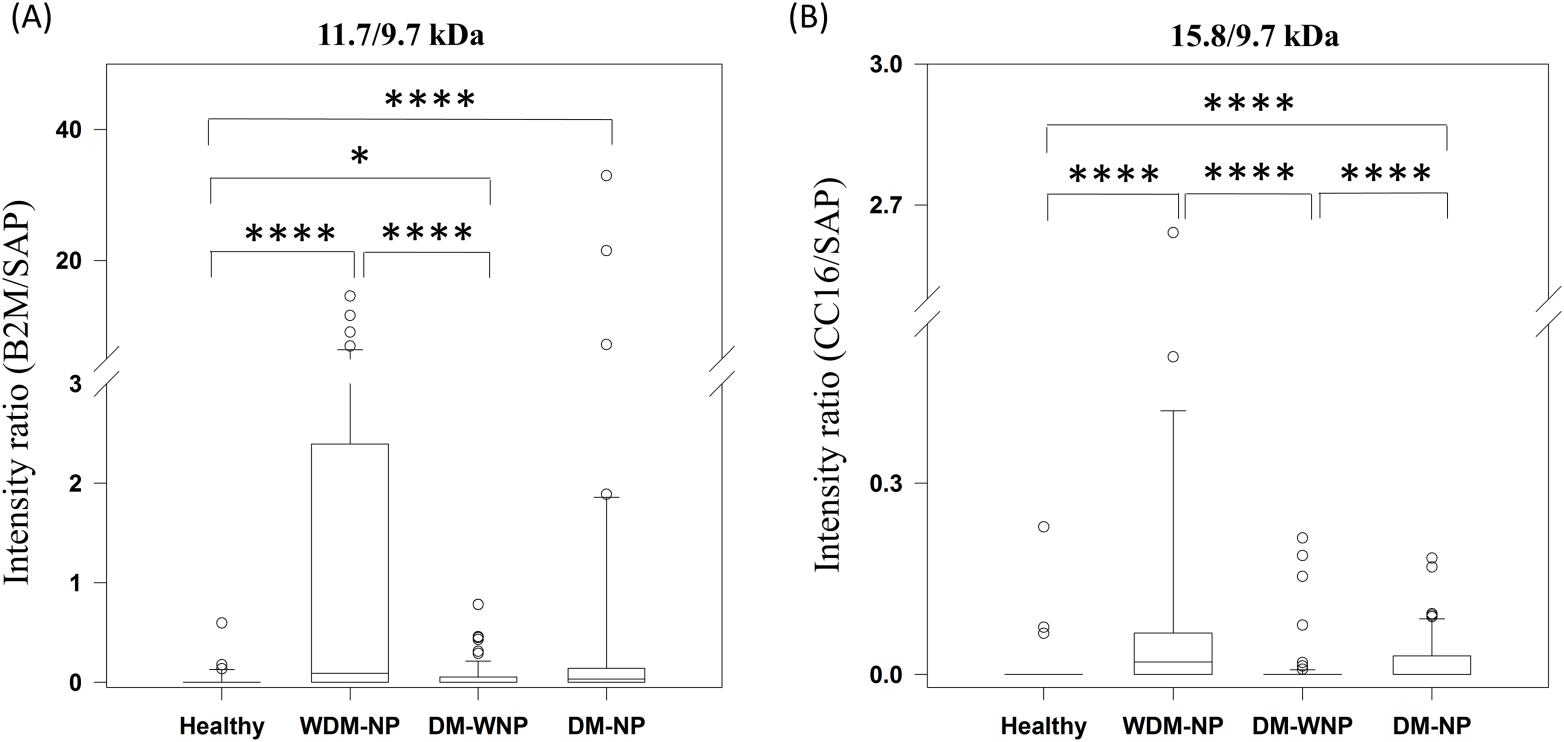
Excretion of (A) 11.7 kDa and (B) 15.8 kDa proteins in urine samples from 39 healthy, 44 WDM-NP, 85 DM-WNP, and 51 DM-NP subjects. The relative intensities are represented as box plots, expressed as the medium with quartile values (25%, 75%). Error bars indicate the minimum and maximum values. * *p* < 0.05, *** *p* ≤ 0.001.

Desalted urinary protein samples from 87 DM-WNP patients, 53 DM-NP patients, 48 WDM-NP patients, and 50 healthy controls were analyzed by MALDI-TOF MS. The representative MALDI-TOF mass spectra from healthy and WDM-NP subjects are shown in Fig 3A. The prominent peaks of *m/z* 11732±2 (or oxidized form *m/z* 11748±2) and *m/z* 15840±3(or oxidized form m/z ~15856±3) were found to be highly expressed in WDM-NP and DM-NP subjects; their representative pseudo-gel and spectrum are shown in Fig 3B. Because the peak of 9.7 kDa (saposine B, SAP) is constantly detected with a strong signals in all samples and not reported as a diabetes or nephropathy marker, SAP was used as the internal standard in this study to evaluate the diagnostic value of the two protein marker peaks of 11.7 kDa and 15.8 kDa. As shown in Fig 4A, the peak ratio of 11.7/9.7 kDa is significantly higher in WDM-NP and DM-NP than in healthy group (p<0.001). The peak ratio of 15.8/9.7 kDa is significantly higher in WDM-NP and DM-NP than in healthy (p<0.001) and DM-WNP groups (p<0.001) (Fig 4B).

To distinguish WDM-NP patients from healthy subjects, the area under the curve (AUC) of the ROC plots was investigated. The AUC was 0.75 for the 11.7 kDa peak and 0.74 for the 15.8 kDa peak. Because these two peaks can be simultaneously examined in a single MALDI-TOF mass spectrum, both can be used as diagnostic markers. These 2 peaks gave a sensitivity and specificity of 77.3% and 91.8%, respectively, with an improved AUC of 0.8 and therefore could be used as markers to discriminate between WDM-NP (nephropathy) and healthy subjects. For the differentiation of DM-NP (diabetic nephropathy) from DM-WNP patients, the AUC was 0.6 for the 11.7 kDa peak and 0.67 for the 15.8 kDa peak. The combined markers of these two peaks in this case had a sensitivity and specificity of 66% and 73% with the AUC of 0.62.

### 3.3 Purification and identification of differentially expressed proteins

**Fig 5:**
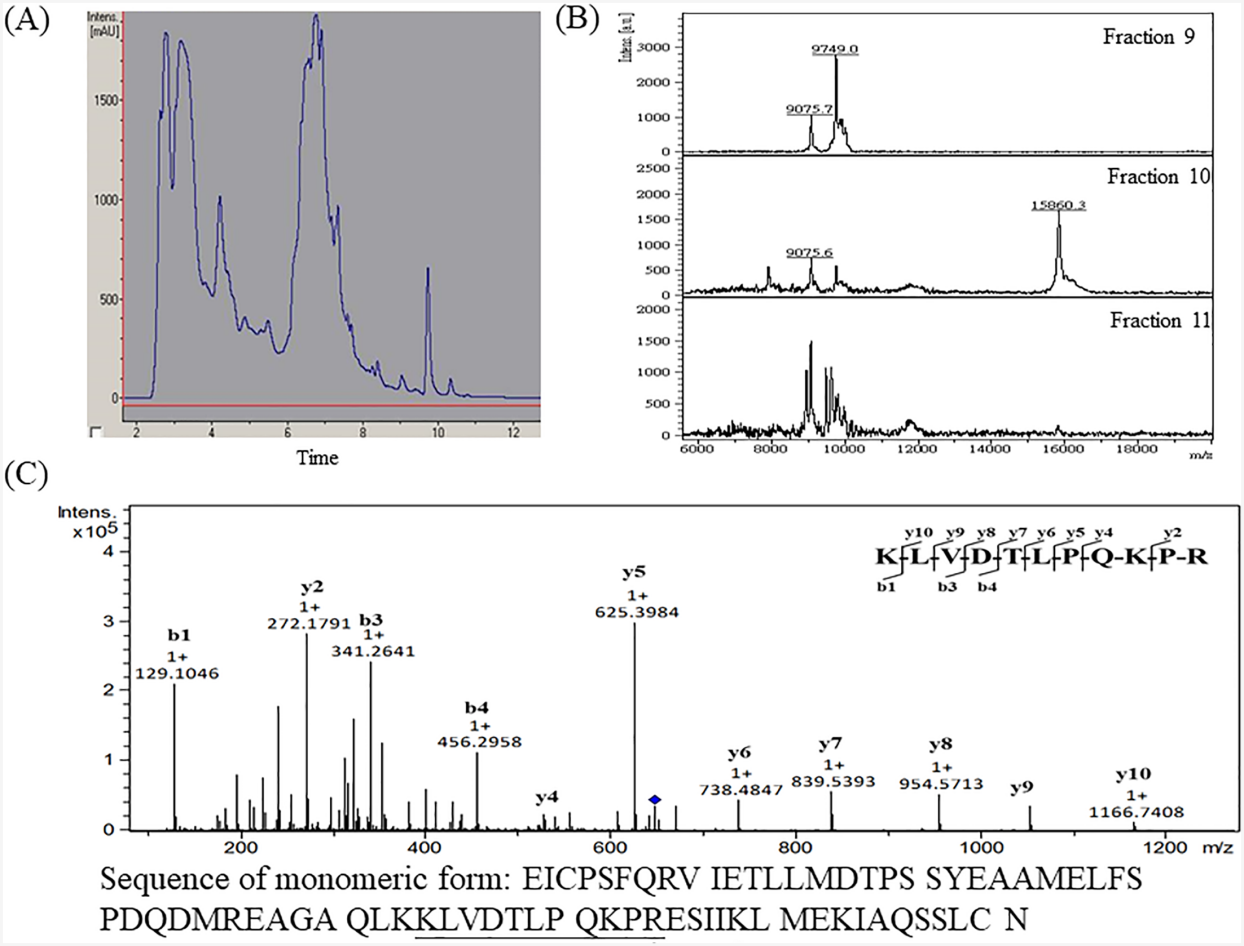
(A) Representative LC-UV chromatogram of urinary proteins from a DM-NP subject. The eluent was collected at 60-s intervals. (B) MALDI-TOF MS analysis of LC subfractions 9, 10, and 11. The marker peak of *m/z* 15860 was found in fraction 10. (C) Identification of the corresponding peptide of the *m/z* 647.91 peak by nanoLC-MS/MS. The sequence of CC16 was shown and the identified peptide sequence was underlined.

The 11.7 kDa peak has been reported to be B2M [14] and was also identified in our previous study [15]. To confirm the identity of the 15.8 kDa peak, the urine samples were fractionated by C_4_ reversed-phase chromatography as described in the Materials and Methods section. The LC-UV chromatogram of urinary proteins from a DM-NP subject is shown in Fig 5A. The 15.8 kDa peak was purified from subfraction 10 (Fig 5B). Subfractions 9, 10, and 11 were separately subjected to in-solution digestion and nanoLC-MS/MS analysis. The 15.8 kDa peak in subfraction 10 was identified as CC16 (Mascot identification score of 88) on the basis of MS/MS sequencing of the doubly charged tryptic peptide peak of *m/z* 647.91, which showed a complete y- and b-ion series corresponding to the sequence KLVDTLPQKPR (Fig 5C).

### 3.4 ELISA evaluation of B2M and CC16

Because ELISA is often used in the clinical laboratory to quantify protein marker abundance for diagnosis, the 11.7 kDa B2M and 15.8 kDa CC16 were subjected to ELISA to evaluate the relative abundance of the two markers in urine. (S3 Fig) B2M expression was significantly higher in the WDM-NP, DM-NP, and DM-WNP groups than in the healthy group (*p* < 0.001). However, CC16 expression was significantly high in the DM-NP group only relative to the DM-WNP (*p* < 0.05) and healthy (*p* < 0.001) groups. To distinguish WDM-NP patients from healthy subjects, the AUC of the ROC plot was evaluated and found to be 0.87 for B2M and 0.67 for CC16, and the combined AUC for B2M and CC16 was 0.74. In the case of distinguishing DM-NP from DM-WNP, the AUC was 0.59 for B2M and 0.60 for CC16, and the combined AUC was 0.60.

### 3.5 Validation of the protein markers in T2D patients who had developed to ERFD

A nested case control study design was used for investigating the prediction ability of B2M and CC16 in the development of nephropathy in T2D patients. The T2D subjects with (n = 56) and without (n = 69) ERFD as primary end point had similarly demographic (age and gender), DM duration, BMI, SBP, DBP, HbA1c, creatinine, and ACR at the baseline examination. Patients with ERFD had higher eGFR than those without ERFD (means ± standard deviations: 114.13 ± 25.86 versus 98.86 ± 23.36 [*p* value = 0.001]). ACR is not a significant marker in baseline for predicting ERFD (*p* value for chi-square test was 0.196 (Table 2)). For the two potential urinary markers, the 16 of 56 (28.6%) T2D patients with ERFD and the 9 of 69 (13.0%) T2D patients without ERFD had detectable CC16 marker (CC16/SAP ratio > 0) at baseline (*p* value for chi-square test was 0.031 (Table 2)). No significant difference between two groups for presence of B2M was observed (39.3% vs. 31.9% for ERFD and non ERFD group, *p* = 0.389). The data showed that the presence of CC16 at baseline is associated with the later development of ERFD.

**Table 2:** Demographic and clinical characteristics stratified by decline in eGFR/year ≧3.3% (ERFD).

	Stable (N = 69)	Rapid decline (N = 56)	*P* value
Age	54.84 (7.98)	56.07 (8.16)	0.399
Male%	40 (58.0%)	30 (54.5%)	0.702
Follow up duration	3.81 (1.88)	3.33 (1.60)	0.124
DM duration	8.80 (7.40)	8.30 (5.88)	0.759
**At haptoglobin measurement**			
HbA1c	7.94 (1.72)	7.92 (1.94)	0.927
Urine Creatinine (mg dL^-1^)	173.62 (136.44)	145.60 (75.35)	0.150
eGFR	98.86 (23.36)	114.13 (25.86)	0.001
BMI	25.63 (3.77)	26.10 (4.11)	0.510
SBP	122.12 (18.51)	127.60 (18.31)	0.102
DBP	71.71 (11.34)	72.62 (10.65)	0.650
**Biomarker concentrations**			
ACR	22.94 (31.25)	33.45 (52.89)	0.196
B2M/SAP_MALDI ratio (>0%)	22 (31.9%)	22 (39.3%)	0.389
CC16/SAP MALDI ratio (>0%)	9 (13.0%)	16 (28.6%)	0.031
**Combine B2M/SAP_MALDI and CC16/SAP MALDI ratio**			
Group1	43 (62.3)	28 (50.0)	0.153
Group2	21 (30.4)	18 (32.1)	
Group3	5 (7.2)	10 (17.9)	

Abbreviation: ACR, albumin to creatinine ratio; SBP, systolic blood pressure; DBP, diastolic blood pressure; eGFR, estimated glomerular filtration rate; BMI, body mass index; ERFD, early renal functional decline; DM, diabetes mellitus. Group1: B2M/SAP_MALDI ratio = 0 and CC16/SAP MALDI ratio = 0. Group2: B2M/SAP_MALDI ratio = 0 and CC16/SAP MALDI ratio > 0, B2M/SAP_MALDI ratio > 0 and CC16/SAP MALDI ratio = 0. Group3: B2M/SAP_MALDI ratio > 0 and CC16/SAP MALDI ratio > 0.

Logistic regression was used to further examine the effect of potential biomarkers, B2M and CC16, independently or combined on ERFD (Table 3). When comparing individuals having marker with those without marker, the OR for ERFD was 2.01 (95% CI: 0.90–4.52) for B2M marker and 4.87 (95% CI: 1.77–13.44) for CC16 marker, respectively after adjusting for follow-up time. Furthermore, there was a significant additive effect of increasing the ERFD risk with combined CC16 and B2M (the *p* value of interaction term = 0.003). The OR for ERFD was 7.59 (95% CI: 1.97–29.24) when comparing individuals having both markers with those without any markers.

**Table 3:** The logistic regression models.

	Basic model			Adjusted model		
	OR	95% CI	*P* value^a^	OR	95% CI	*P* value^b^
**B2M/SAP_MALDI ratio**						
0	Ref.	Ref.		Ref.	Ref.	
>0	1.38	0.66–2.89	0.390	2.01	0.90–4.52	0.090
**CC16/SAP MALDI ratio**						
0	Ref.	Ref.		Ref.	Ref.	
>0	2.67	1.07–6.62	0.035	4.87	1.77–13.44	0.002
**Combine B2M/SAP_MALDI and CC16/SAP MALDI ratio**						
Group1	Ref.	Ref.		Ref.	Ref.	
Group2	1.32	0.60–2.90	0.495	1.95	0.82–4.66	0.134
Group3	3.07	0.95–9.94	0.061	7.59	1.97–29.24	0.003

^a^ *P* value for logistic regression model, unadjusted

^b^ *P* value for logistic regression model adjusted for follow up duration

Group1: B2M/SAP_MALDI ratio = 0 and CC16/SAP MALDI ratio = 0. Group2: B2M/SAP_MALDI ratio = 0 and CC16/SAP MALDI ratio > 0, B2M/SAP_MALDI ratio > 0 and CC16/SAP MALDI ratio = 0. Group3: B2M/SAP_MALDI ratio > 0 and CC16/SAP MALDI ratio > 0.

## 4. Discussion

Recently, MALDI-TOF MS has successfully approved as an in vitro diagnostic device for routine bacterial identification in hospitals [16]. Therefore, disease markers detected by MALDI-TOF MS is getting practical for clinical use. Because salts in urine can interfere with the MALDI-TOF mass spectral signals, in this study, a C18 plate was used to rapidly remove salts from clinical samples and retain urinary proteins in, urine samples for MALDI-TOF MS analysis. Our results showed that CC16 was more highly expressed in the WDM-NP and DM-NP groups than in the healthy (*p* < 0.001) and DM-WNP groups (*p* < 0.001). However, in the ELISA results, CC16 expression was only slightly higher in the DM-NP group than in the DM-WNP group (*p* = 0.05). ELISA results may be affected by the specificity of antibody; however, MALDI-TOF MS have high specificity in detecting the protein markers by measuring their protein mass. The above results also indicate that MALDI-TOF MS can identify CC16 more specifically than ELISA assay and could be a better approach with respect to the reliability of diagnosis.

B2M is a low-molecular-weight protein that is filtered by the glomerulus and degenerated in the proximal tubules [17]. Some studies have shown that urinary B2M increases in renal tubular injuries, suggesting urinary B2M to be an early diagnostic marker of tubular injury [18, 19]. In type 2 diabetes, urinary B2M excretion has been associated with macrovascular disease [20] and nephropathy [1, 21, 22].

The 15.8-kDa CC16 (also known as CC10, uteroglobin, or urinary protein 1) was sequenced by Jackson et al.[23] and Bernard et al.[24] and identified as a protein composed of two subunits of 7.8 kDa connected by disulfide bonds with a measured mass of ~15839.5±2.1 Da.[25] CC16 is rapidly eliminated by glomerular filtration, and reabsorbed and catabolized in the renal proximal tubule cells. Dysfunction of the proximal tubule cells can cause diminished resorption of CC16 and its increased levels in urine. CC16 has been reported as a marker of proximal tubular dysfunction in adult [26] and child patients [27]. This protein marker is sensitive to very subtle defects in proximal tubular dysfunction that may not be detected by assay of classical urinary low-molecular-weight proteins [28]. To the best of our knowledge, our study is the first to find high CC16 expression in urine of patients with nephropathy and DN.

With a long term follow-up study, the B2M and CC16 (OR of 7.59 for developing ERFD) were found to be independent predictors for ERFD among T2D patients who had not yet manifested significant kidney disease at baseline and indicated that the protein peaks of B2M and CC16 detected by C18 plate/MALDI-TOF may improve the sensitivity for predicting nephropathy before the appearance of urinary albumin.

Although the CC16 and B2M share the same mechanisms of glomerular filtration and tubular reabsorption, there is still some difference between the two markers. Our ELISA results shows that the median concentration of B2M (7.8 μg/mg creatinine) in urine of healthy subjects is ~116-fold higher than CC16 (0.067μg/mg creatinine), and the median concentration of B2M (26.9 μg/mg creatinine) in urine of WDM-NP (nephropathy) subjects is ~302-fold higher than CC16 (0.089μg/mg creatinine). B2M protein signals is also significantly stronger than CC16 in our MALDI-TOF results. Therefore, B2M is easier to be detected than CC16. However, CC16 is more specific and sensitive in the diagnosis of diabetes nephropathy than B2M, because its AUC is higher than B2M in the discrimination between diabetes and diabetes nephropathy. Also, the OR of CC16 (4.87) is higher than B2M (2.01) in predicting risk of the development of nephropathy in diabetes groups.

## 5. Conclusions

From a large-sample size analysis, we discovered and validated 2 protein peaks, B2M (11.7 kDa) and CC16 (15.8 kDa), as biomarkers associated with nephropathy and verified the discriminatory ability in a set of 238 individuals including diabetic and nondiabetic patients. The OR of combined B2M and CC16 markers for developing ERFD was 7.59 (95% CI: 1.97–29.24). This is the first report of CC16 as a urinary marker of nephropathy and DN. Our approach of detecting B2M and CC16 by C_18_ plate—MALDI-TOF MS may thus provide rapid diagnosis and prediction of nephropathy in type 2 diabetes patients.

## Supporting information

S1 Fig MALDI-TOF mass spectra of urinary protein amount of 0.6 μg (top panel), 1.19 μg (middle panel), and 2.38 μg (bottom panel) from a patient with micro- or macroalbuminuria due to nondiabetic disease. (TIF)

S2 Fig Box plots of the total protein concentration in urine samples from 46 healthy, 47 WDM-NP, 87 DM-WNP, and 53 DM-NP subjects. (TIF)

S3 Fig ELISA assay of (A) β2-microglobulin (B2M) and (B) Clara-cell protein (CC16) in urine samples from 39 healthy, 44 WDM-NP, 85 DM-WNP, and 51 DM-NP subjects. (TIF)

## Abbreviations

B2M: β2-microglobulin
CC16: Clara-cell protein
DM: diabetes mellitus
DM-NP: patients with type 2 diabetes mellitus with microalbuminuria
DM-WNP: patients with type 2 diabetes mellitus without micro- or macroalbuminuria
DN: diabetic nephropathy
MALDI-TOF-MS: matrix-assisted laser desorption/ionization time-of-flight mass spectrometry
SA: Sinapic acid
SELDI-TOF-MS: surface-enhanced laser desorption/ionization time-of-flight mass spectrometry
WDM-NP: patients with micro- or macroalbuminuria due to nondiabetic disease
