# Supplementary figures and images for: Urine proteome analysis by C18 plate–matrix-assisted laser desorption/ionization time-of-flight mass spectrometry allows noninvasive differential diagnosis and prediction of diabetic nephropathy
Source: PLoS One. 2018 Jul 19;13(7):e0200945. doi: 10.1371/journal.pone.0200945 (PMC6053209; doi:10.1371/journal.pone.0200945)

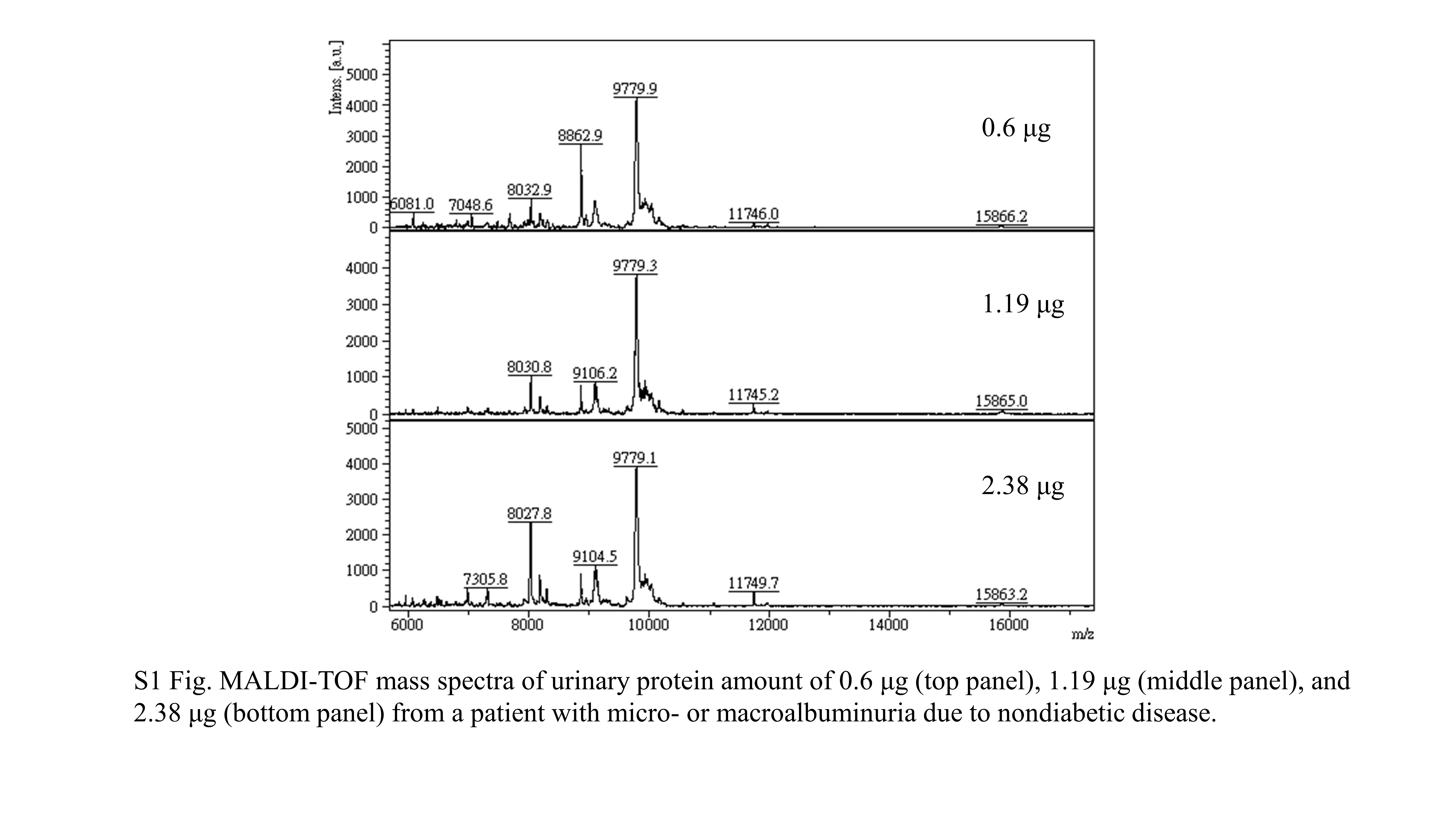

Supplement: S1 Fig — (TIF) [file pone.0200945.s001.tif]

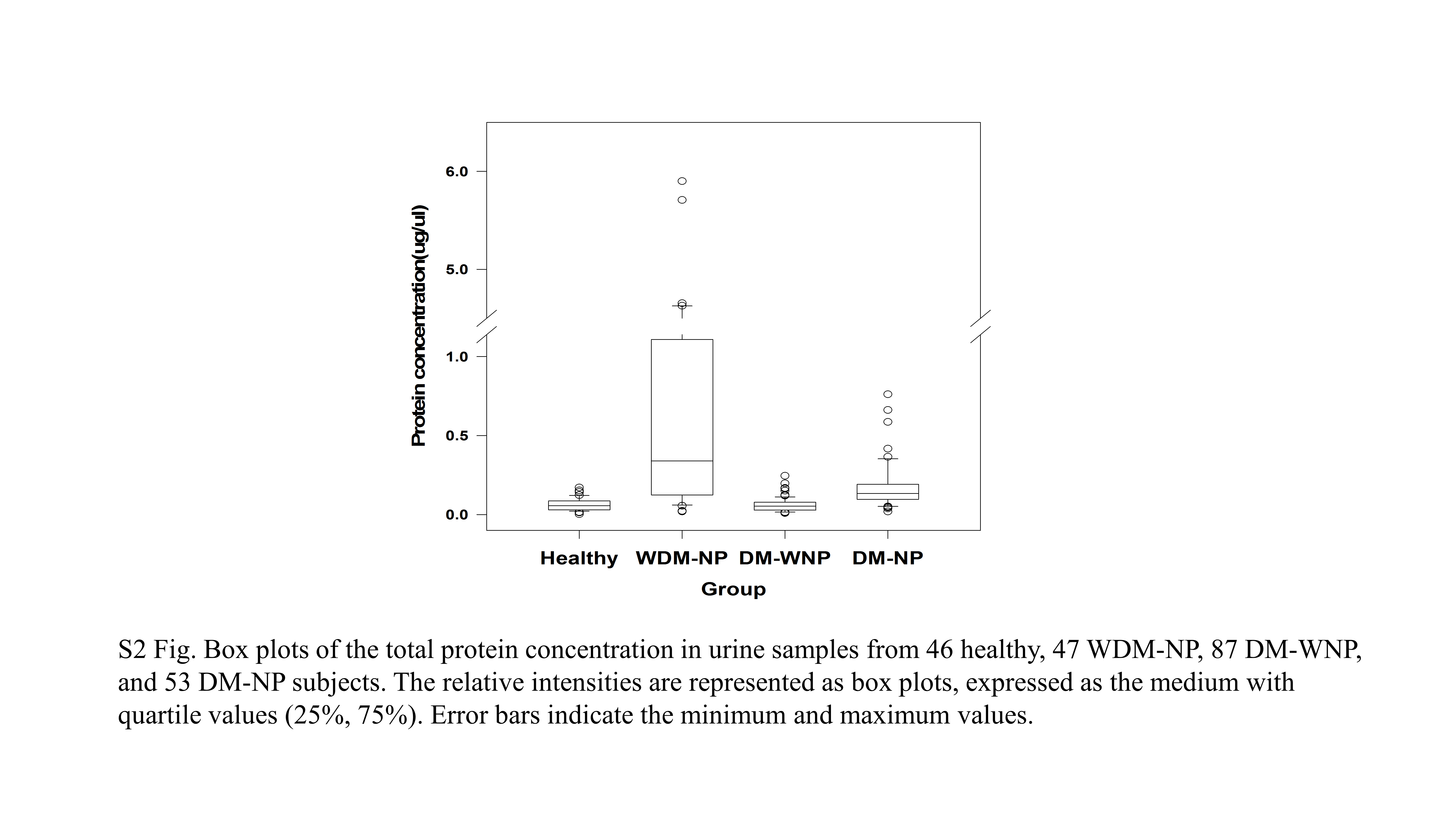

Supplement: S2 Fig — (TIF) [file pone.0200945.s002.tif]

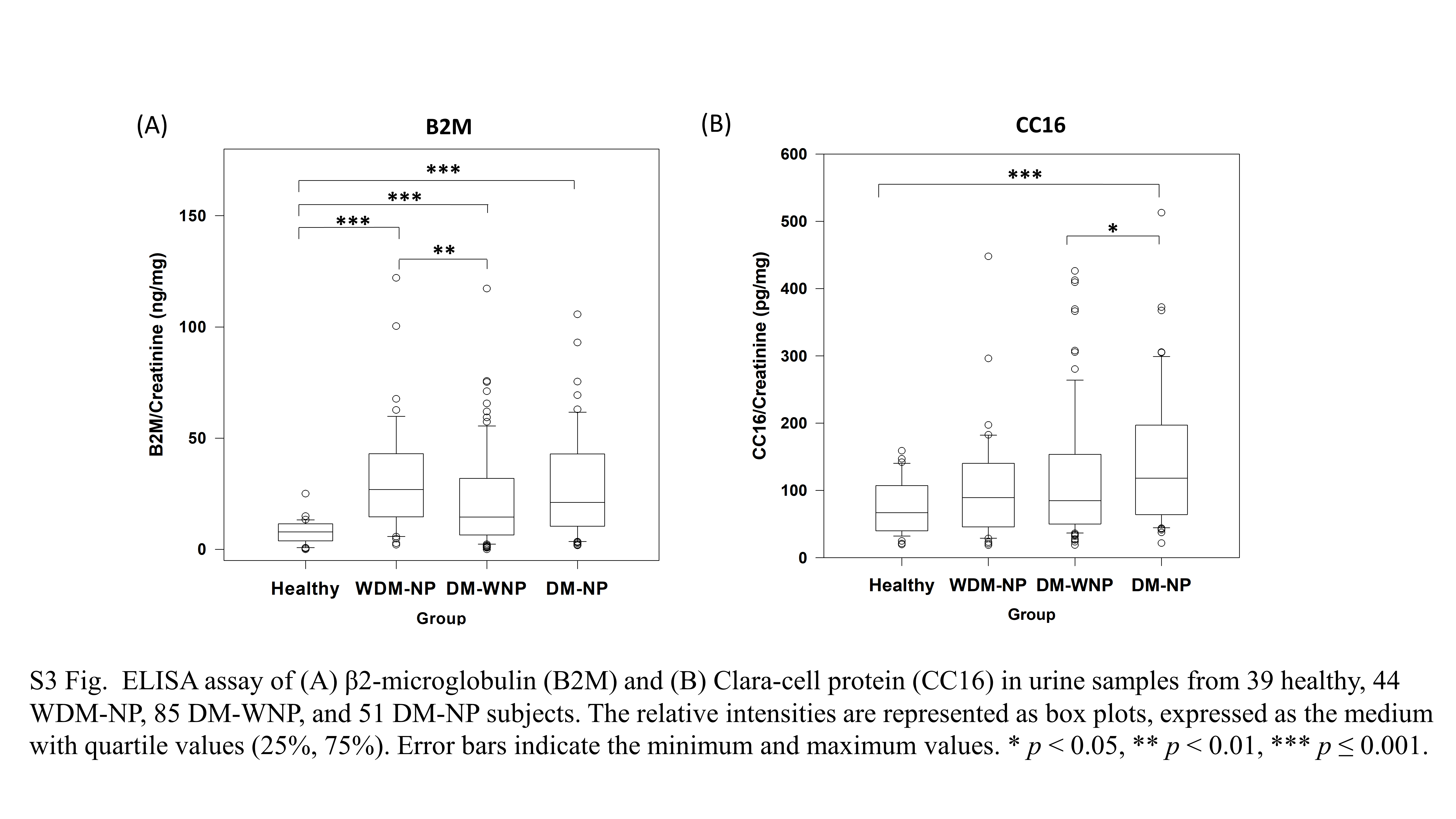

Supplement: S3 Fig — (TIF) [file pone.0200945.s003.tif]
